# Risk prediction in MDS: independent validation of the IPSS-M—ready for routine?

**DOI:** 10.1038/s41375-023-01831-1

**Published:** 2023-02-01

**Authors:** Constance Baer, Sandra Huber, Stephan Hutter, Manja Meggendorfer, Niroshan Nadarajah, Wencke Walter, Uwe Platzbecker, Katharina S. Götze, Wolfgang Kern, Torsten Haferlach, Gregor Hoermann, Claudia Haferlach

**Affiliations:** 1 MLL Munich Leukemia Laboratory, Max-Lebsche-Platz 31, 81377 Munich, Germany; 2 Medical Clinic and Policlinic 1, Hematology and Cellular Therapy, University of Leipzig, Leipzig, Germany; 3 Technical University of Munich (TUM), School of Medicine, Department of Internal Medicine III, Munich, Germany

**Keywords:** Myelodysplastic syndrome, Risk factors, Cancer genetics

Myelodysplastic neoplasms (MDS) are clonal disorders of hematopoietic cells characterized by peripheral cytopenias, morphologic dysplasia, ineffective hematopoiesis and risk of leukemic transformation (LT) [1, 2]. Biology and clinical outcome within MDS are extremely heterogeneous, making individual risk prediction key for management and therapy decision. Prognostic scoring systems are used for risk prediction in MDS, and the current Revised-International Prognostic Scoring System (IPSS-R) is based on clinical variables and cytogenetic aberrations [3]. Next generation sequencing (NGS) identified recurrently mutated genes, and molecular data have been used to refine the prognostication in MDS [4–6]. However, there has been no commonly accepted standard for incorporation of NGS data into established prognostic scoring systems. The new Molecular International Prognostic Scoring System (IPSS-M) includes the mutation status of 31 genes in addition to cytogenetics, bone marrow blasts, hemoglobin level, and platelet count [7]. In total, the IPSS-M requires 37 parameters including the *TP53* multihit status *(TP53*^multi^; combination of mutations, deletion or copy neutral loss of heterozygosity (CN-LOH)), *KMT2A* and *FLT3* aberrations as well as a pattern of co-mutations for *SF3B1*. The IPSS-M provides a continuous patient-specific risk score grouped into six risk categories, defined as very low (VL), low (L), moderate low (ML), moderate high (MH), high (H) and very high (VH) [7].

To perform an independent validation of the IPSS-M, we selected 626 *de novo* MDS patients, who were referred to our laboratory between 09/2005 and 01/2020 (Supplementary Table S1) with a median follow-up of 9.5 years. Data was obtained from peripheral blood and bone marrow using cytomorphology, cytogenetics and molecular genetics as described [8–10]. Patients were genetically characterized in-depth by amplification-free whole genome sequencing (WGS), in addition to routine work-up. With a median coverage > 100x, WGS is able to achieve a sensitivity of 10–15% variant allele frequency [11]. CN-LOH was assessed using HadoopCNV [12]. The IPSS-R was calculated for 452 cases with available data on absolute neutrophil counts, which is not necessary for calculation of the IPSS-M.

Our real-world cohort of 626 MDS patients showed a distribution among the six IPSS-M risk categories of 15% VL, 41% L, 11% ML, 7% MH, 12% H and 14% VH (Fig. 1A). The observed risk distribution is well comparable with the initial publication of the IPSS-M also showing a skewing towards low-risk categories [7]. We also found a clear prognostic separation for overall survival (OS), leukemia free survival (LFS) and LT according to IPSS-M categories (Fig. 1B) which is comparable with the initial publication but shows a slightly worse discrimination e.g., of the MH risk category probably due to the lower number of patients in this group (*n* = 42).

**Fig. 1:**
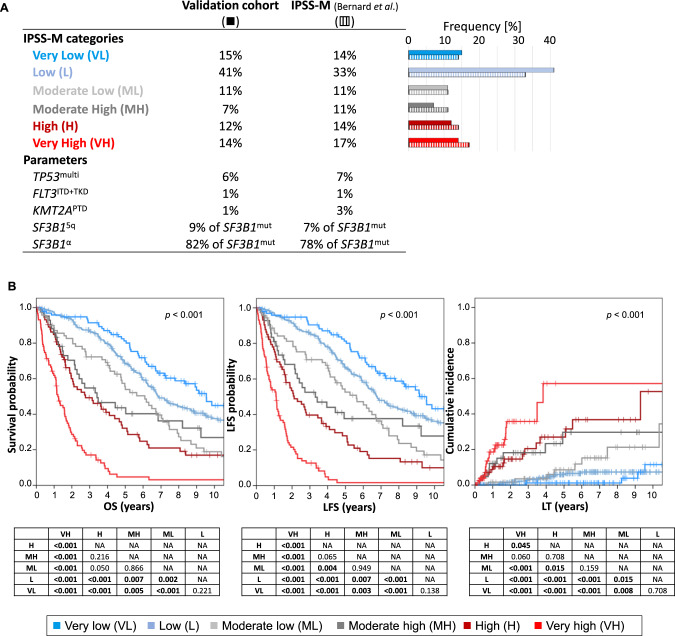
Validation of the IPSS-M. **A** Frequencies of IPSS-M categories and specific parameters within our MDS validation cohort (*n* = 626) compared to the data published by Bernard et al. **B** Kaplan–Meier plots for MDS patients classified according to the IPSS-M for overall survival (OS), leukemia free survival (LFS) and leukemic transformation (LT).

We then compared the individual risk categorization according to IPSS-R and IPSS-M in 452 patients (Fig. 2A). When considering the IPSS-M categories ML and MH combined as one group, 111 patients (25%) were up- and 87 patients (19%) down-staged according to IPSS-M. For the majority of patients (38%), the risk group differed only by one level (up-stage: 90; down-stage: 80), while larger differences of more than one level were observed in 6% of patients (up-stage: 21; down-stage: 7). Exploratory analysis of patients up- or down-staged more than one level suggests that the new IPSS-M category better reflects the individual survival (Supplementary Fig. S1). For a systematic approach, we used the *Harrell’s* concordance index (c*-*index [13]) to assess the correlation between predictions according to the IPSS-R and IPSS-M with real outcomes. The c-index for the IPSS-R was 0.68 (OS), 0.69 (LFS) and 0.77 (LT), and improved to 0.71 (OS), 0.73 (LFS) and 0.81 (LT) for the IPSS-M (Fig. 2B). Our results closely match the values of the original IPSS-M publication and highlight the benefit of incorporating molecular genetic variables.

**Fig. 2:**
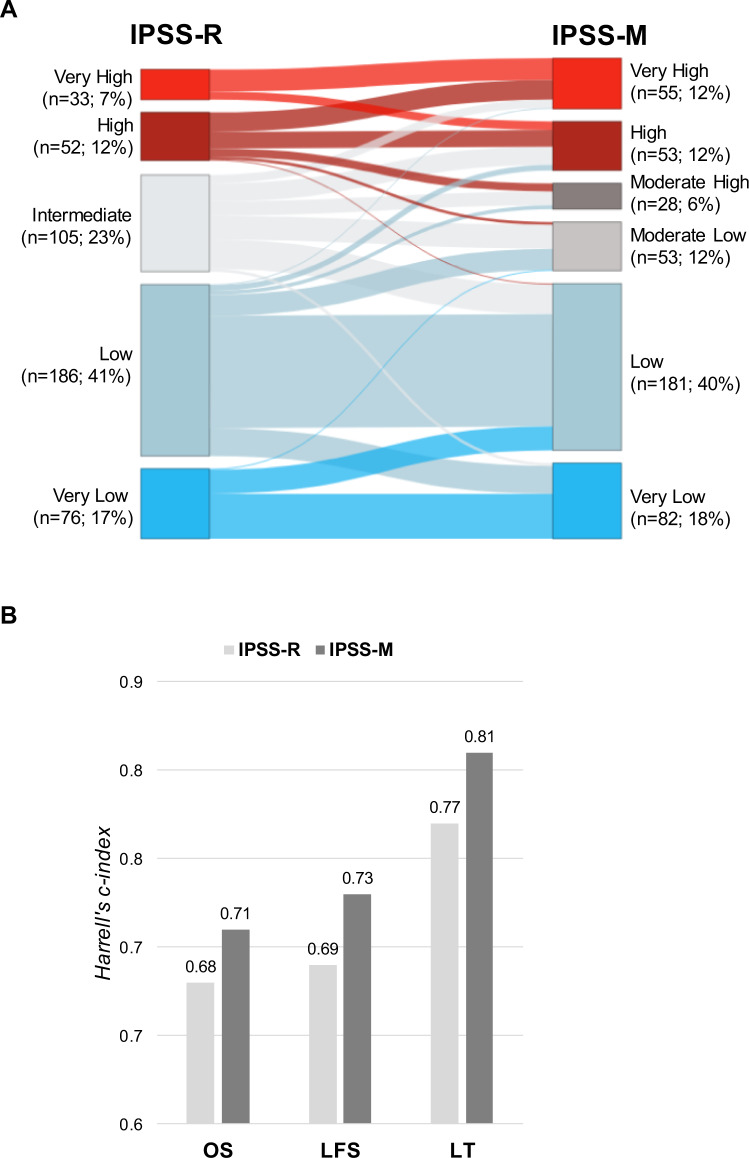
Comparison of IPSS-R and IPSS-M. **A** Changes in MDS risk categories of MDS patients (*n* = 452) according IPSS-R and IPSS-M. **B** Model discrimination as measured by *Harrell’s c-index* of the IPSS-R or IPSS-M across the different end points (OS overall survival, LFS leukemia-free survival, LT leukemic transformation).

Finally, we focused on the relative importance of individual genes for risk prediction according to the IPSS-M and underlying practical aspects. In daily practice, missing values and the finding of genetic variants of unknown significance, which cannot clearly be classified as mutated or unmutated, are challenging. In general, the IPSS-M is able to handle missing values and calculates best- and worst-case scenarios. According to IPSS-M*, TP53*^multi^*, FLT3*^ITD+TKD^ and *KMT2A*^PTD^ are the top predictors of adverse outcomes. We identified 67 patients with *TP53* mutations (11%) of whom 35 (6%) fulfill the status of *TP53*^multi^ (7 with two mutations, 16 with mutation(s) + deletion, and 12 with mutation + CN-LOH). Conventional panel sequencing does not necessarily allow to analyze the CN-LOH status, and a high variant allele frequency (VAF; e.g., >55%) has been proposed as a surrogate. In our cohort, only one patient (mutation with 46% VAF + CN-LOH) would have been missed for *TP53*^multi^ status without CN-LOH analysis indicating that high VAF could be a valuable surrogate for *TP53*^multi^ in daily practice. Furthermore, we found *FLT3*^ITD+TKD^ in 7 (1%; all VH) and *KMT2A*^PTD^ in 6 cases (1%; 2 VH, 3 H, 1 ML). *KMT2A*^PTD^ poses a specific challenge since current targeted NGS panels typically do not allow the assessment of the underlying large intragenic duplications. In order to gauge the effect of missing *KMT2A*^PTD^ status we specifically left out this parameter for mutated patients: One patient remained in the VH group independently of the *KMT2A*^PTD^ status, while for the other patients the best- and worst- case scenarios led to sometimes vastly different categories (Supplementary Table S2). Since imprecise categories are not useful in daily practice, a determination of *KMT2A*^PTD^ status is necessary for the use of the IPSS-M although it is a rare aberration in MDS (1% here and 2.5% in the IPSS-M cohort). If lower weighted variables, e.g., the “residual genes”, would be not available, this resulted in 31% of cases displaying consistent categories between best- and worst-case scenarios (Supplementary Fig. S2).

In addition, we identified *NPM1* mutations, which are also integrated into the IPSS-M model, in 4 cases (1%; 1 VH, 3 H). However, based on the 5th edition of WHO Classification *NPM1* mutated patients are now categorized as AML with mutated *NPM1* irrespective of the blast count [2]. As the prognostic impact of *SF3B1* mutations (*SF3B1*^mut^) depends on their co-abnormalities, the IPSS-M discriminates *SF3B1* mutations in the presence of isolated del(5q) (*SF3B1*^5q^) and *SF3B1* mutations without specific co-mutations (*SF3B1*^α^). Within all *SF3B1* mutated cases (*n* = 199), *SF3B1*^5q^ was found in 9% and *SF3B1*^α^ in 82% of samples (Supplementary Fig. S3).

In conclusion, we independently confirm the increased predictive power of the IPSS-M in MDS as compared to IPSS-R. We used a real-world cohort of 626 patients diagnosed at a single center with profound genetic analysis using WGS. In both our data set and the original IPSS-M cohort, the c-index was lowest for OS. This could be explained by the fact that age is not taken into account. Age per se is a risk factor for OS and associated with other comorbidities. Two other recently published molecular MDS scores include age with moderate [14] or strong weight [15]. To illustrate the effect of age, we looked at the youngest and oldest 10th percentile. While both subgroups have about a quarter of patients in the IPSS-M H or VH group (*n* = 11/43 vs. 9/43), their predicted OS probability at 60 months according to the above mentioned scores is very different (54 vs. 36% [14] and 76 vs. 28% [15], Supplementary Fig. S4). In line with the age-adjusted IPSS-R [3], age adjustment of the IPSS-M may improve the prediction of OS. In contrast, LT and LFS are more influenced by the biology of the disease and the genetic aberrations of the MDS clone already included in the IPSS-M. While all new molecular risk prediction tools for MDS have been shown to be superior to the IPSS-R [7, 14, 15], the objective of the given tool should be tailored to the application (e.g., personal therapeutic decision making or clinical study design).

In daily practice, we use both a classification (homogeneous morphology or genetics) and prognosis systems (homogeneous risk but possibly different biology). Ultimately, those are two sides of the same coin, which only combined lead to the best (targeted) therapeutic solutions. In summary, a comprehensive molecular analysis has become new standard for prognostication of patients with MDS. On a short term, not all laboratories will be able to offer broad molecular genetic panels with all 31 genes of the IPSS-M. In addition to conventional targeted NGS, complementary genetic methods (like PCR and FISH) and/or specifically designed and validated NGS assays are needed to reliably assess *TP53*^multi^*, FLT3*^ITD^ and *KMT2A*^PTD^. The ability of the IPSS-M online calculator to handle missing data will facilitate implementation into routine diagnostics. However, results of such an approach are only clinically meaningful if the spectrum from best case to worst case scenarios is narrow enough to allow an acceptable risk assessment. For highly weighted variables (e.g., *KMT2A*-PTD) this will rarely be the case. This makes laboratory analysis for MDS more relevant than ever. It requires different laboratory branches and clinicians to join forces to yield a complete set of all types of data allowing a meaningful prognostic score for state-of-the-art management of MDS patients in 2023+.

## Supplementary information

Supplements

## Data Availability

The datasets generated during and/or analyzed during the current study are available from the corresponding author on reasonable request.
