# Supplementary material for: Risk prediction in MDS: independent validation of the IPSS-M—ready for routine?
Source: Leukemia. 2023 Feb 1;37(4):938–41. doi: 10.1038/s41375-023-01831-1 (PMC10079546; doi:10.1038/s41375-023-01831-1)
Supplement: Supplementary file 1 — Supplements [file 41375_2023_1831_MOESM1_ESM.pdf]

Supplemental Material

Supplementary Figure S1. Overall survival **(A)** and leukemia free survival **(B)** time from diagnosis to death based on the IPSS-R risk categories; dots represent patients and their survival within each risk categories (only patients who died are included). Purple dots show patients who were up-staged by the IPSS-M, blue dots show patients who were down-staged by the IPSS-M. VL, very low; L, low; I, intermediate; H, high; VH, very high; OS, overall survival; LFS, leukemia free survival.

A

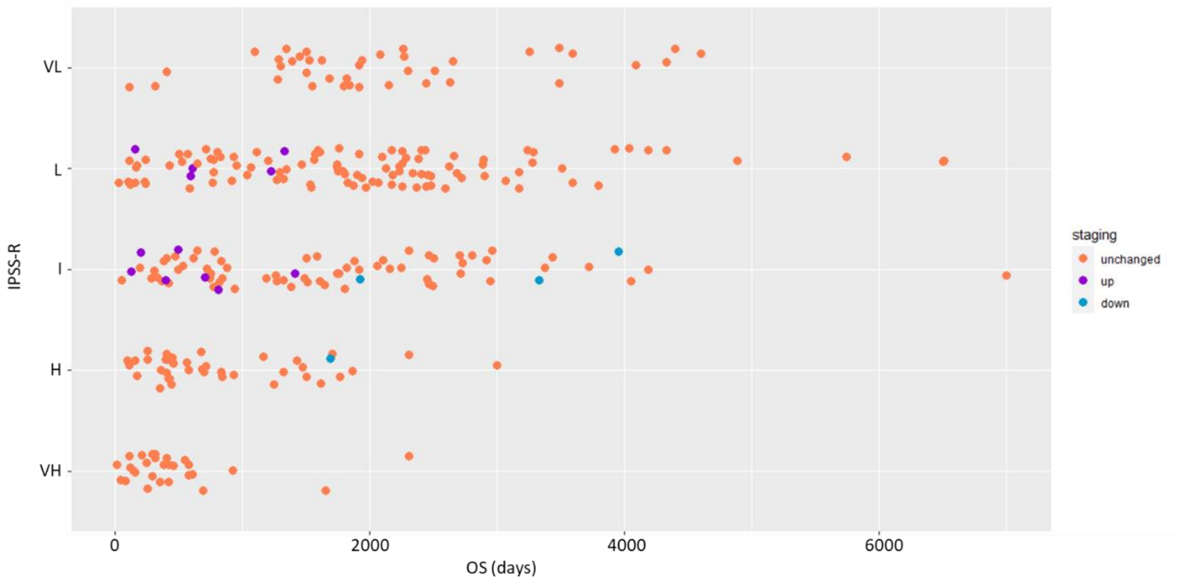

B

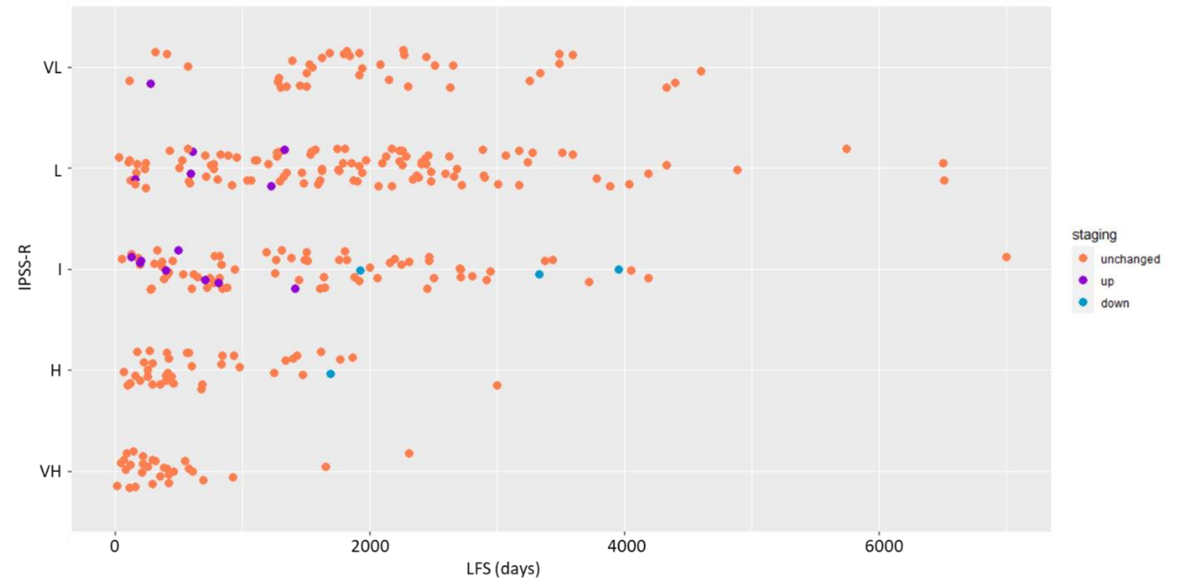

Supplementary Figure S2. Effect of missing residual gene information on differences in best/worst-case IPSS-M scores (line/dots) and categories (bars). One by one genes where cumulatively removed from the data set until all 15 residual genes were removed from analysis. The order of removal was determined by the mutational frequency reported in the original IPSS-M publication from most to least mutated (*STAG2* to *PRPF8*, upper panel) and from least to most mutated (*PRPF8* to *STAG2*, lower panel).

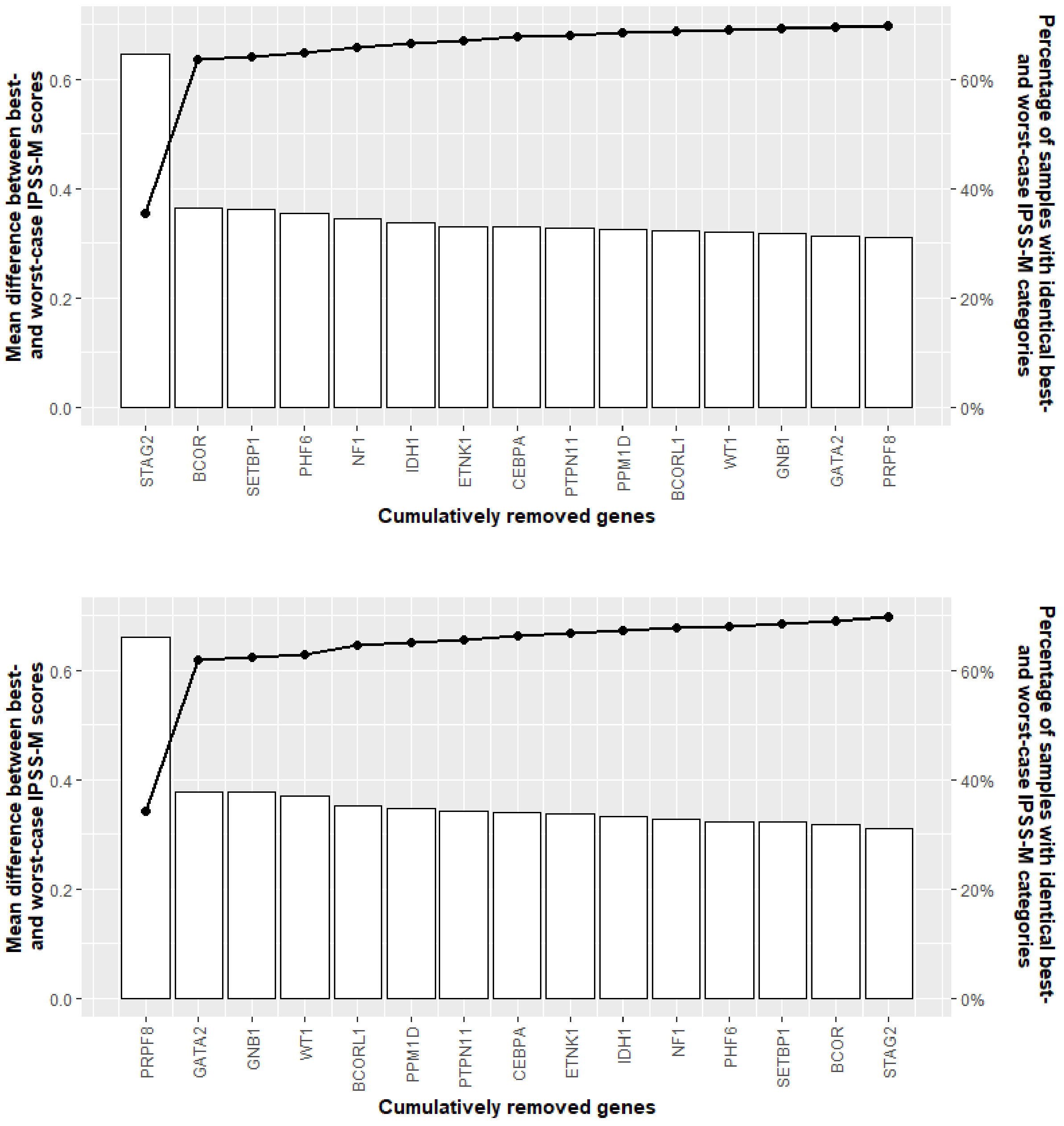

Supplementary Figure S3. Kaplan-Meier analysis for overall survival (OS) according to different *SF3B1* categories, namely *SF3B1*^{5q}, *SF3B1*$^{\alpha}$ (without co-mutations in *BCOR, BCORL1, RUNX1, NRAS, STAG2, SRSF2*) and *SF3B1*$^{\beta}$ (with corresponding co-mutations).

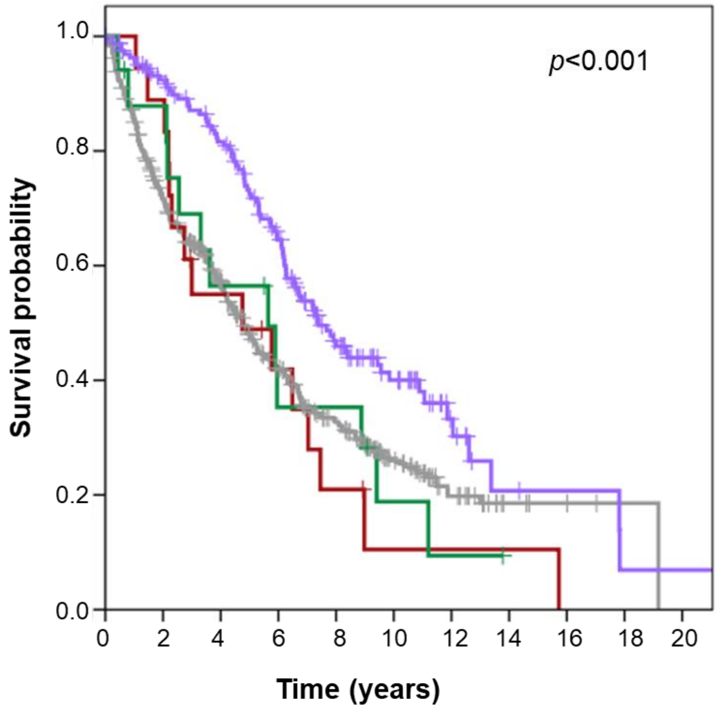

***SF3B1* category**	**n**	**Median OS (years)**
SF3B1$^{\alpha}$	164	7.4
SF3B1$^{\beta}$	17	5.6
SF3B1^{5q}	18	4.8
SF3B1$^{wild\text{-}type}$	427	4.8
**Total**	**626**	**5.7**

Supplementary Figure S4. The patients are sorted in ascending order by age. The youngest and oldest 10% are highlighted (n=43). For these, the OS probability after 60 months was calculated according two scores, which include age.[1,2]

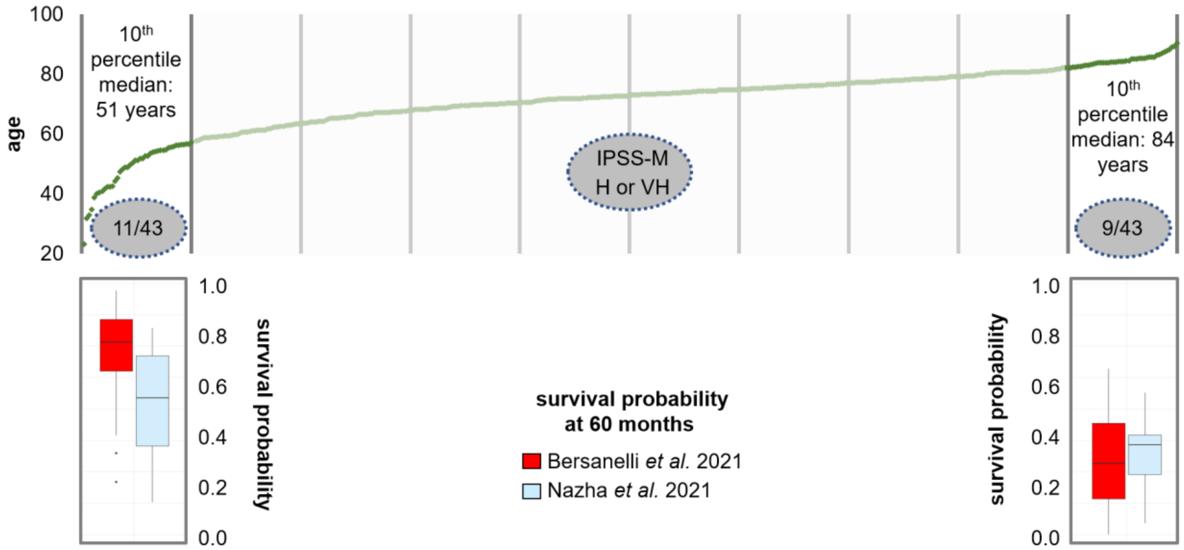

1. Nazha A, Komrokji R, Meggendorfer M, Jia X, Radakovich N, Shreve J, et al. Personalized Prediction Model to Risk Stratify Patients With Myelodysplastic Syndromes. Journal of Clinical Oncology. 2021;39(33):3737-46.
2. Bersanelli M, Travaglino E, Meggendorfer M, Matteuzzi T, Sala C, Mosca E, et al. Classification and Personalized Prognostic Assessment on the Basis of Clinical and Genomic Features in Myelodysplastic Syndromes. Journal of Clinical Oncology. 2021;39(11):1223-33.

Supplementary Tables

Suppl. Table S1. MDS cohort overview

**Characteristics**	**MDS cases (n = 626)**
**Age** (years; median [range])	73 [23-93]
**Sex** (female / male)	265 (42%) / 361 (58%)
**Karyotype** (normal / aberrant)	367 (59%) / 259 (41%)
**Cytogenetic risk group**	**Number of samples (%)**
Very poor	40 (6.5)
Poor	27 (4)
Intermediate	57 (9)
Good	462 (74)
Very good	40 (6.5)
**WHO 2017 diagnosis**	
MDS with single lineage dysplasia (MDS-SLD)	20 (3)
MDS with multilineage dysplasia (MDS-MLD)	97 (16)
MDS with single lineage dysplasia with ring sideroblasts (MDS-RS-SLD)	47 (8)
MDS with multilineage dysplasia with ring sideroblasts (MDS-RS-MLD)	128 (20)
MDS with isolated del(5q) (MDS 5q-)	87 (14)
MDS with excess blasts (MDS-EB-1)	120 (19)
MDS with excess blasts (MDS-EB-2)	127 (20)

Suppl. Table S2. IPSS-M scores and categories in best/worst case scenarios for patients with *KMT2A*^{PTD}

**Sample #**	**with *KMT2A*^{PTD} worst case scenario**	**without *KMT2A*^{PTD} best case scenario**
1	2.88 (VH)	1.72 (VH)
2	2.58 (VH)	1.44 (H)
3	0.98 (H)	- 0.17 (ML)
4	0.75 (H)	- 0.40 (ML)
5	0.55 (H)	- 0.61 (L)
6	- 0.20 (ML)	- 1.35 (L)
